# Exploring the carbon footprint of severe asthma and change after biologic therapy initiation: an analysis of Northern Irish data

**DOI:** 10.1183/23120541.01009-2024

**Published:** 2025-06-23

**Authors:** John Busby, Mina Khezrian, Soram Patel, Trung N. Tran, Kirsty Rhodes, Liam G. Heaney

**Affiliations:** 1 School of Medicine, Dentistry and Biomedical Sciences, Queen's University Belfast, Belfast, UK; 2 Biopharmaceuticals Medical, AstraZeneca, Cambridge, UK; 3 Biopharmacetuicals Medical, AstraZeneca, Gaithersburg, MD, USA; 4 Belfast Health and Social Care NHS Trust, Belfast, UK

## Abstract

**Background:**

The carbon footprint of severe asthma and the impact of biologic therapy in this population is unknown.

**Methods:**

This was a retrospective cohort study in adults with severe asthma, using data from the Northern Ireland Regional Severe Asthma Service (September 2015–November 2021). We calculated annual greenhouse gas (GHG) emissions (carbon dioxide equivalent) for asthma-related medications and healthcare resource utilisation, compared patient characteristics by GHG quartile, calculated GHG change post-biologic initiation, and explored the relationship between GHG change and clinical response.

**Results:**

Among 303 patients with severe asthma, mean±sd GHG emissions were 474±431 kg, largely driven by SABA use (50.7%) and emergency department (ED) visits/inpatient admissions (21.0%). Those with highest-quartile GHG emissions were more likely to have uncontrolled disease (Asthma Control Questionnaire-5 score 3.5 *versus* 2.5; p<0.001), be more deprived (46.1% *versus* 25.0%; p=0.029) and have depression/anxiety (35.5% *versus* 14.7%; p=0.002) *versus* those with lowest-quartile GHG emissions. Among patients who received a biologic (n=213), modest GHG reductions (−28.0±286 kg; p=0.15) were observed, largely driven by a reduction in ED/hospitalisation-related GHG emissions (−59.3±224 kg; p<0.001). SABA-related GHG emissions were relatively unchanged (−6.1±138 kg; p=0.518). Total GHG emissions were 72.4±352 kg (p=0.044) lower than baseline at 4 years post-biologic initiation. Although there was substantial clinical improvement post-biologic initiation, this was not associated with GHG reductions.

**Conclusions:**

Severe asthma is associated with substantial GHG emissions, primarily driven by SABA use and emergency care utilisation. Although GHG emissions were lower post-biologic, largely due to a reduction in emergency care, the changes in GHG emissions were modest and SABA use was relatively unchanged. An improved understanding of the factors driving elevated GHG emissions is required.

## Shareable abstract

**Severe asthma is associated with substantial GHGs mainly due to SABA and emergency care use. GHG emissions were lower post-biologic largely due to a reduction in emergency care, but changes were modest and SABA use was relatively unchanged.** https://bit.ly/3C1d30c

## Introduction

According to the World Health Organization, climate change presents a fundamental threat to human health, describing it as “a threat multiplier, undermining and potentially reversing decades of health progress” [1]. Reducing greenhouse gas (GHG) emissions has become increasingly important to governments worldwide as the economic, political and health consequences of climate change become apparent [2]. Within the UK, healthcare contributes 4–5% of total GHG emissions [3], representing a key target for climate change mitigation efforts. Consequently the National Health Service (NHS) has set a target of “Net Zero” by 2040 [4], with an ambition for an 80% reduction (compared with a 1990 baseline) by 2028 to 2032 [4, 5]. Northern Ireland is committed to this target, Wales is committed to a 90% reduction by 2050 and Scotland is committed to “Net Zero” by 2045 [5].

Respiratory diseases are a major contributor to GHG emissions due to routine inhaled medications and acute exacerbations [6, 7]. Within the NHS, inhalers account for 13% of emissions [8], with asthma care contributing an estimated 750 kilotonnes carbon dioxide equivalent (CO_2_e) annually [7]. Around 5% of the UK's asthma population have severe asthma [9, 10], which drives much of the morbidity and treatment-related burden of asthma [11, 12]. An increasing number of biologic therapies have become available for severe asthma, with compelling experimental and real-world evidence of their effectiveness leading to discussion of whether clinical remission is now an achievable treatment goal.

To date, studies have focused on the carbon footprint of asthma in a broader asthma population managed within UK primary care, and on the impact of inhalers on GHG emissions [7, 13–18]. No data exist on the environmental impact of biologics in severe asthma. This study uses real-world data from Northern Ireland to describe the magnitude and correlates of GHG emissions among patients with severe asthma. We quantified change in GHG emissions after biologic initiation and explored whether there was a correlation between GHG change and measures of clinical response.

## Methods

### Study design and data sources

This was a single-arm cohort study based on routinely collected medical data extracted from the Northern Ireland Regional Severe Asthma Service. On attending clinic, patients had their asthma systematically assessed by specialists and data collected on their demographic, clinical and asthma treatment. Data on therapeutic response were collected at annual review visits. Ethics approval was obtained from the Health and Social Care Research Ethics Committee A (reference number: 290836).

### Study population

Patients attended the clinic between September 2015 and November 2021, were biologic-naive at initial clinical assessment, and met European Respiratory Society/American Thoracic Society criteria [19] for severe asthma at their initial visit.

Patients entered the study at the date of their first asthma medication (*i.e.* short-acting β_2_-agonist (SABA), inhaled corticosteroid, long-acting β_2_-agonist, theophylline, long-acting muscarinic antagonist, leukotriene receptor antagonist and/or oral corticosteroid (OCS)). The index date for patients receiving biologics was the biologic start date, while for those who did not receive biologics it was the date of first clinic attendance. Patients exited the cohort on 2 December 2022, when information on biologic use was extracted. All patients were followed up for a minimum of 1 year and a maximum of 4 years.

Patients were excluded if the start date of their biologic was unknown, they entered the study <1 year prior to their index date, they exited the study <1 year after their index date or if there was no clinical data available at index (defined as within 60 days).

### GHG emissions quantification

GHG emission calculations were based on the Sustainable Health Coalition's Care Pathway Guidance and methodology (supplementary material S1) [20], categorised as either medication- or healthcare resource utilisation (HCRU)-related, and expressed as CO_2_e. Emissions were quantified using SimaPro life cycle assessment software modelling resource and energy consumption data, Ecoinvent datasets, certified published studies [18, 21, 22], and modelled estimates. Medication-related emissions included those from the entire life cycle of inhalers, including manufacture and transport of the inhaler device (*e.g.* pressurised metered-dose inhaler (pMDI) or dry powder inhaler (DPI)), and end-of-life disposal. HCRU-related emissions included those attributed to asthma-related outpatient visits, emergency department (ED) visits and hospitalisations, and included an allowance for patient travel. No data were available on primary care consultations.

### Study variables

Definitions and timings of assessment of key HCRU and asthma variables collected pre- and post-biologic are provided in table 1 with further details provided in supplementary material S1.

**TABLE 1:** Definition or method of assessment and timing of assessment for key demographic, healthcare resource utilisation (HCRU) and asthma clinical variables collected

Variable	Definition or method of assessment	Source	Pre-index	Post-index
**Demographics**				
MDM	Patient socioeconomic status measured by quintile of the 2017 Northern Ireland MDM	NISRA	NA	NA
Demographics	Patient age, sex, ethnicity, BMI, smoking status, comorbidities	NIRSAS	At index	NA
**HCRU outcomes**				
Asthma exacerbation	Acute asthma episode requiring rescue systemic corticosteroids	NIRSAS	1 year pre-index	Annualised post-index
Asthma ED attendance	Diagnosis contained the word “asthma”	Secondary care records	1 year pre-index	Annualised post-index
Asthma hospitalisation	Primary diagnosis of asthma (ICD-10 code: J45)	Secondary care records	1 year pre-index	Annualised post-index
Outpatient visit	Specialty of “Thoracic Medicine” or “Thoracic Surgery”	Secondary care records	1 year pre-index	Annualised post-index
**Clinical outcomes**				
Spirometry	Conducted according to ERS/ATS guidelines and percentage predicted values using the GLI 2012 multi-ethnic reference values	NIRSAS	At index	Annual reviews post-index
Asthma control	Measured using the ACQ (five-question version: ACQ-5)	NIRSAS	At index	Annual reviews post-index
HRQoL	Measured using the EuroQol EQ-5D-5L instrument	NIRSAS	At index	Annual reviews post-index
Biomarkers	Type 2 biomarkers (BEC, *F*_ENO_, IgE-mediated)	NIRSAS	At index	Annual reviews post-index
SABA use	Objective: number of dispensed canisters	Dispensing data	1 year pre-index	Annual reviews post-index
	Subjective: through patients’ response to Question 6 of the ACQ: “On average, during the past week, how many puffs of short-acting bronchodilator have you used each day?”	NIRSAS	At index	Annual reviews post-index
mOCS use	Daily mOCS use: dose expressed as prednisolone equivalent (mg)	NIRSAS	At index	Annual reviews post-index
Asthma medications	Asthma add-on therapies	NIRSAS	At index	NA
Biologic therapy	Start and stop dates for all biologic therapies	NIRSAS	NA	NA
**Composite outcomes**				
Super-response	ACQ-6 improvement ≥0.5 (or well-controlled (ACQ-6 ≤0.75) at annual review), AND exacerbation reduced ≥50% (or no exacerbations in the 12 months prior to annual review), AND unscheduled care reduction ≥50% (or no unscheduled care in the 12 months prior to annual review), AND FEV_1_ improvement ≥100 mL, and OCS dose reduction ≥50% (or not a mOCS user at annual review)	NIRSAS	At index	Annual reviews post-index
Clinical remission	No evidence of poor symptom control (ACQ-5 <1.5), no exacerbations in the previous 12 months AND no mOCS at the time of first annual review	NIRSAS	At index	Annual reviews post-index

MDM: multiple deprivation measure; NISRA: Northern Ireland Statistics and Research Agency; NA: not applicable; BMI: body mass index; NIRSAS: Northern Ireland Regional Severe Asthma Service; ED: emergency department; ICD: International Classification of Diseases; ERS: European Respiratory Society; ATS: American Thoracic Society; GLI: Global Lung Function Initiative; ACQ: Asthma Control Questionnaire; HRQoL: health-related quality of life; BEC: blood eosinophil count; *F*_ENO_: exhaled nitric oxide fraction; SABA: short-acting β_2_-agonist; (m)OCS: (maintenance) oral corticosteroid; FEV_1_: forced expiratory volume in 1 s.

### Statistical methods

We calculated medication-related, HCRU-related and overall GHG emissions in the year prior to the index date for the overall population. To investigate factors that may be associated with GHG emissions we compared the baseline demographic and clinical characteristics by overall GHG quartiles. Descriptive statistics are presented as means with standard deviation, medians (interquartile range (IQR)) or counts (percentages), depending on data distribution. Univariable analyses were conducted using t-tests, Chi-squared tests or Mann–Whitney U-tests as appropriate. For each patient who initiated a biologic, we calculated GHG emissions in the years post-biologic initiation and compared the mean GHG emissions to that for the pre-biologic period using paired samples t-tests. We explored the relationship between relative GHG change and measures of clinical response (asthma symptoms (Asthma Control Questionnaire (ACQ)-5), exacerbations, lung function (percentage predicted forced expiratory volume in 1 s (FEV_1_)) and health-related quality of life (HRQoL; assessed by the EuroQol EQ-5D-5L instrument)) using Spearman's correlation. We compared GHG change by super-response and remission status using independent samples t-tests (see table 1 for definitions). Analyses were conducted using Stata 16 (StataCorp, College Station, TX, USA). Outputs are consistent with the Office for National Statistics disclosure guidelines [23].

### Sensitivity and supplementary analysis

To better understand the drivers of GHG emissions among biologic-treated patients, we categorised GHG emissions in the year post-biologic initiation (overall, medication- and HCRU-related) and compared demographic and clinical characteristics by GHG quartile as described earlier. To prevent selection bias, we repeated our analysis of GHG change after biologic initiation restricted to patients with at least 4 years of follow-up. We repeated our analysis using Mann–Whitney U-tests (change from baseline) and Wilcoxon signed-rank tests (comparison by composite response) to ensure that hypothesis tests were robust to departures from normally distributed data.

## Results

### Cohort description

Data were available on 355 patients, of whom 303 were eligible for analysis (supplementary material S2). Of these, 213 (70.3%) received a biologic, with follow-up data available for 213 (100.0%), 199 (93.4%), 154 (72.3%) and 98 (46.1%) patients at 1, 2, 3 and 4 years post-initiation, respectively. Patient demographic and clinical characteristics are provided in tables 2 and 3. Patients exhibited substantial morbidity, including frequent exacerbations (median 4), high symptom burden (mean ACQ-5 score 3.0) and impaired lung function (mean FEV_1_ 70.3% predicted). They had high SABA use in the prior year (median 10 cannisters dispensed, 5.8 puffs per day self-reported) and 62.7% were receiving maintenance OCS at index. Mepolizumab (90.1%) was the most commonly prescribed biologic at baseline, with most (86%) continuing with their initially prescribed biologic for at least 1 year (supplementary material S3). The demographic and clinical characteristics of those progressing to biologics were similar to the overall cohort (supplementary material S4 and S5).

**TABLE 2:** Demographics, comorbidities and healthcare resource utilisation at index by carbon quartile for the total severe asthma population at baseline

	All patients	GHG quartile				p-value
		1 (lowest)	2	3	4 (highest)	
**Patients**	303	75	76	76	76	
**GHG emissions (kg CO_2_e)**	474.4±431.5	112.3±44.7	255.3±45.5	464.8±87.9	1060.6±457.9	<0.001
**Demographics**						
Female	183 (60.4)	39 (52.0)	42 (55.3)	49 (64.5)	53 (69.7)	0.096
Age (years)	51.5±14.5	56.1±11.1	51.3±15.3	49.9±15.3	48.8±14.9	0.011
Asthma duration (years)	25.7±16.4	24.5±17.2	25.9±16.7	25.9±15.9	26.5±16.1	0.901
Caucasian	303 (100.0)	75 (100.0)	76 (100.0)	76 (100.0)	76 (100.0)	
Deprivation tertile						0.029
1 (most)	104 (34.7)	18 (25.0)	30 (39.5)	21 (27.6)	35 (46.1)	
2	102 (34.0)	24 (33.3)	21 (27.6)	31 (40.8)	26 (34.2)	
3 (least)	94 (31.3)	30 (41.7)	25 (32.9)	24 (31.6)	15 (19.7)	
BMI (kg·m^−2^)	31.4±7.1	31.4±7.8	31.3±7.3	30.2±6.1	32.6±6.9	0.213
Obese (>30 kg·m^−2^)	159 (52.8)	38 (51.4)	42 (55.3)	39 (51.3)	40 (53.3)	0.956
Ever-smoker	123 (40.7)	24 (32.0)	32 (42.1)	35 (46.1)	32 (42.7)	0.329
Atopic disease	98 (45.2)	23 (41.8)	21 (39.6)	30 (51.7)	24 (47.1)	0.575
**Comorbidities**						
Depression or anxiety	62 (20.5)	11 (14.7)	11 (14.5)	13 (17.1)	27 (35.5)	0.002
GORD	61 (20.1)	11 (14.7)	<13.2	22 (28.9)	19 (25.0)	0.024
Nasal polyps	56 (18.5)	14 (18.7)	23 (30.3)	10 (13.2)	<13.2	0.014
**HCRU (prior year)**						
Exacerbations	4 (2–6)	4 (2–5)	4 (2–6)	4 (3–6)	6 (3–10)	0.002
Any asthma ED attendance	70 (23.1)	<13.3	11 (14.5)	16 (21.1)	38 (50.0)	<0.001
Any asthma hospitalisation	74 (24.4)	<13.3	10 (13.2)	19 (25.0)	43 (56.6)	<0.001
Outpatient visits	7 (4–11)	6 (3–9)	7 (4–12)	8 (6–12)	9 (6–13)	0.003

Data are presented as n, mean±sd, n (%) or median (interquartile range), unless otherwise stated. GHG: greenhouse gas; CO_2_e: carbon dioxide equivalent; BMI: body mass index; GORD: gastro-oesophageal reflux disease; HCRU: healthcare resource utilisation; ED: emergency department.

**TABLE 3:** Clinical measures and asthma medications at index by carbon quartile for the total severe asthma population at baseline

	All patients	GHG quartile				p-value
		1 (lowest)	2	3	4 (highest)	
**Patients**	303	75	76	76	76	
**GHG emissions (kg CO_2_e)**	474.4±431.5	112.3±44.7	255.3±45.5	464.8±87.9	1060.6±457.9	<0.001
**Lung function**						
FEV_1_ (%)	70.3±21.1	70.9±18.1	72.0±24.0	70.9±19.5	67.4±22.4	0.568
FVC (%)	87.1±18.3	88.0±16.9	87.9±20.9	87.6±15.9	84.9±19.2	0.693
FEV_1_/FVC	64.5±12.6	64.2±12.4	65.1±13.0	65.2±12.8	63.6±12.2	0.854
**Patient-reported outcomes**						
ACQ-5 score	3.0±1.4	2.5±1.3	2.9±1.3	3.2±1.3	3.5±1.5	<0.001
EQ-5D-5L utility score	0.70 (0.44–0.89)	0.80 (0.67–0.92)	0.74 (0.44–0.92)	0.68 (0.48–0.89)	0.51 (0.31–0.83)	0.004
**Type 2 biomarkers**						
BEC (×10^9^ L^−1^)	0.34 (0.14–0.56)	0.31 (0.13–0.58)	0.28 (0.15–0.54)	0.39 (0.14–0.60)	0.36 (0.16–0.56)	0.871
Highest BEC (×10^9^ L^−1^)	0.75 (0.56–1.13)	0.77 (0.59–1.12)	0.79 (0.52–1.10)	0.71 (0.55–1.24)	0.73 (0.57–1.15)	0.978
*F*_ENO_ (ppb)	36 (19–63)	39 (16–64)	39 (19–68)	32 (22–64)	36 (20–54)	0.967
IgE (IU·mL^−1^)	132 (45–416)	132 (34–328)	117 (46–356)	140 (55–557)	136 (48–373)	0.626
**Asthma medications**						
Daily ICS dose (BDP equivalent) (μg)	2000 (1600–2000)	2000 (1600–2000)	2000 (1600–2000)	2000 (1800–2000)	2000 (1600–2000)	0.733
SABA inhalers	10 (4–18)	4 (1–7)	6 (4–10)	12 (9–17)	24 (16–34)	<0.001
Daily SABA puffs	5.8±4.7	3.5±3.4	5.2±4.3	7.1±4.7	7.5±5.3	<0.001
pMDI SABA	213 (75.3)	27 (45.8)	55 (75.3)	>65 (>86.37)	64 (84.2)	<0.001
Maintenance OCS	188 (62.7)	49 (67.1)	49 (64.5)	42 (56.0)	48 (63.2)	0.540
Daily dose (mg)	10 (8–10)	10 (6–10)	10 (8–10)	10 (5–15)	10 (10–15)	0.097
LAMA	96 (41.2)	17 (27.9)	21 (36.8)	29 (48.3)	29 (52.7)	0.027
Theophylline	73 (31.5)	19 (31.1)	19 (33.3)	12 (20.3)	23 (41.8)	0.102
LTRA	125 (53.6)	32 (52.5)	29 (50.9)	36 (60.0)	28 (50.9)	0.717
Macrolides	25 (8.4)	<13.9	<13.3	<13.2	<13.2	0.447
Nebuliser	49 (21.8)	11 (18.3)	<18.9	12 (20.3)	18 (34.0)	0.090
Biologic progression	213 (70.3)	53 (70.7)	57 (75.0)	52 (68.4)	51 (67.1)	0.727

Data are presented as n, mean±sd, median (interquartile range) or n (%), unless otherwise stated. GHG: greenhouse gas; CO_2_e: carbon dioxide equivalent; FEV_1_: forced expiratory volume in 1 s; FVC: forced vital capacity; ACQ-5: Asthma Control Questionnaire-5; EQ-5D-5L: EuroQol EQ-5D-5L instrument; BEC: blood eosinophil count; *F*_ENO_: exhaled nitric oxide fraction; ICS: inhaled corticosteroid; BDP: beclometasone dipropionate; SABA: short-acting β_2_-agonist; OCS: oral corticosteroid; LAMA: long-acting muscarinic antagonist; LTRA: leukotriene receptor antagonist.

### Magnitude and correlates of GHG emissions in the year prior to index

Among 303 patients with severe asthma, mean±sd GHG emissions in the year prior to index were 474±432 kg. There was wide variation across patients, with 32 (10.6%) having GHGs <100 kg and 27 (8.9%) having GHGs >1000 kg. The majority of GHG emissions were driven by medications (66.5% of total), specifically SABA usage (50.7% of total; 76.3% of medications total). HCRU accounted for 33.5% of GHG emissions, with ED visits/inpatient admissions (21.0% of total) driving most of this.

In the year prior to index, patients in the highest quartile of GHG emissions tended to be younger (mean 48.8 *versus* 56.1 years; p=0.011) than those in the lowest quartile, although average duration of disease was similar (26.5 *versus* 24.5 years; p=0.901) (table 2). They were also more likely to reside in a deprived area (most deprived tertile: 46.1% *versus* 25.0%; p=0.029), but there was no evidence of differences in the proportion with obesity (53.3% *versus* 51.4%; p=0.956) or having ever smoked (42.7% *versus* 32.0%; p=0.329). In general, comorbidities were more likely among those with higher GHG emissions, particularly depression or anxiety (35.5% *versus* 14.7%; p=0.002) and gastro-oesophageal reflux (25.0% *versus* 14.7%; p=0.024). Those in the highest quartile of GHG emissions reported a larger number of exacerbations (6 *versus* 4; p=0.004) and were more likely to have attended the ED (50.0% *versus* <13.3%; p<0.001) or been admitted to hospital (56.6% *versus* <13.3%; p<0.001) for their asthma in the prior year (table 2). They also reported greater asthma symptoms (ACQ-5 score 3.5 *versus* 2.5; p<0.001) and poorer HRQoL (EQ-5D-5L utility score 0.51 *versus* 0.80; p=0.004), although there was no evidence of differences in the median blood eosinophil count (0.36 *versus* 0.31×10^9^ L^−1^; p=0.871) or exhaled nitric oxide fraction (36 *versus* 39 ppb; p=0.967). They had substantially higher SABA use, measured using both canisters dispensed (24 *versus* 4; p<0.001) and self-reported puffs per day (7.5 *versus* 3.5; p<0.001), and were more likely to be using pMDI formulations (84.2% *versus* 45.8%; p<0.001) (table 3).

### Change in clinical outcomes after biologic initiation

Among the 213 patients receiving a biologic, there was substantial clinical improvement in the year following initiation (supplementary material S6). ACQ-5 score improved by a median (IQR) of 0.6 (0.0–1.4), exacerbations reduced by a median (IQR) of 3 (1–5) and FEV_1_ increased by a median (IQR) of 50 (−190–300) mL. Most patients (87.8%) reduced their maintenance OCS dose. Despite these improvements, residual morbidity remained at 1 year post-biologic initiation with a substantial proportion still reporting uncontrolled symptoms (60.3%; ACQ-5 score >1.5), impaired lung function (41.2%; FEV_1_ <80% predicted) or required asthma-related hospitalisation (7.7%). There was no change in median (IQR) self-reported SABA use at 1 year post-biologic initiation (0.0 (−3.5–0.0)).

### Change in GHG emissions after biologic initiation

**FIGURE 1:**
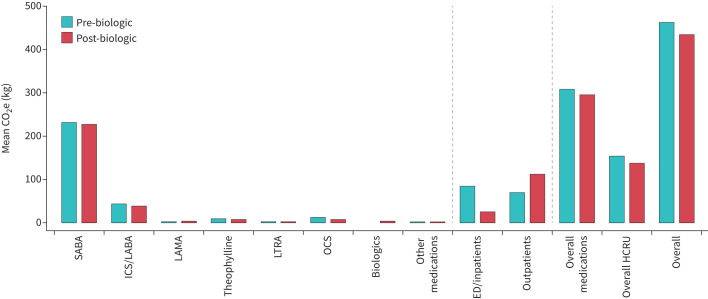
Greenhouse gas emissions in the year pre- and post-biologic for those who initiated a biologic (n=213). CO_2_e: carbon dioxide equivalent; SABA: short-acting β_2_-agonist; ICS: inhaled corticosteroid; LABA: long-acting β_2_-agonist; LAMA: long-acting muscarinic antagonist; LTRA: leukotriene receptor antagonist; OCS: oral corticosteroid; ED: emergency department; HCRU: healthcare resource utilisation.

Among the 213 patients receiving a biologic, mean GHG emissions reduced by 28.0 kg (p=0.154) from 462.2 to 434.3 kg in the first year following biologic initiation (figure 1 and supplementary material S7). Medication-related GHG emissions reduced by 11.4 kg (p=0.298). There was a substantial decrease of 4.4 kg (p<0.001) in OCS from 12.9 to 8.4 kg, but only a small reduction of 6.1 kg (p=0.518) observed for SABAs from 234.3 to 228.2 kg, despite this driving much of the medication-related GHG burden. Healthcare-related GHG emissions reduced by 16.6 kg (p=0.297) from 154.8 to 138.2 kg. Although there was a substantial reduction in ED/inpatient admission GHG emissions of 59.3 kg (p<0.001), this was largely offset by an increase of 42.7 kg in outpatient GHG emissions (p<0.001).

**FIGURE 2:**
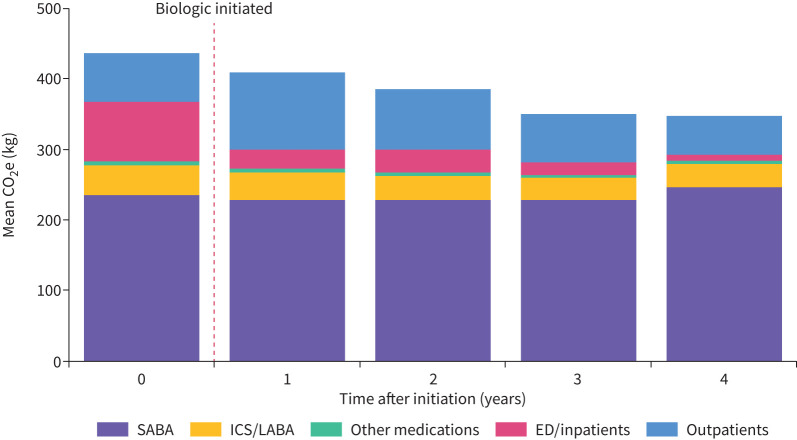
Greenhouse gas emissions in the year pre-biologic and the 4 years post-biologic by carbon source for those who initiated a biologic (n=213). CO_2_e: carbon dioxide equivalent; SABA: short-acting β_2_-agonist; ICS: inhaled corticosteroid; LABA: long-acting β_2_-agonist; ED: emergency department.

Mean GHG emissions continued to reduce in the 4 years post-initiation by 45.5 kg (p=0.037), 104.3 kg (p<0.001) and 72.4 kg (p=0.044) at 2, 3 and 4 years post-biologic initiation, respectively (figure 2 and supplementary material S7). These reductions were primarily driven by a substantial decrease in outpatient-related GHG emissions, falling from 112.8 kg in the year following biologic initiation to 56.9 kg at 4 years post-biologic initiation. ED/inpatient-associated emissions, although contributing modestly to total emissions, reduced from 25.3 to 7.4 kg during the same time frame. There was no evidence of a decrease in SABA-related GHG emissions at 4 years post-biologic initiation (p=0.620).

### Relationship between GHG change and clinical response

No relationship was found between change in GHG emissions in the year following biologic initiation and change in exacerbations (r= −0.01, p=0.855), symptoms (r=0.03, p=0.683), FEV_1_ (r=0.00, p=0.990) or HRQoL (r=0.01, p=0.944) (supplementary material S8). Overall, 34 (19.4%) and 20 (10.6%) patients met criteria for super-response and remission, respectively, at 1 year post-biologic initiation. There was no evidence of a greater decrease in GHG emissions within these groups (supplementary material S9).

### Sensitivity and supplementary analyses

Those with the highest GHG emissions in the year following biologic initiation tended to be more deprived (most deprived tertile: 40.7% *versus* <20.4%; p=0.039), ever-smokers (51.9% *versus* 26.5%; p=0.022), have higher rates of depression or anxiety (27.8% *versus* <20.4%; p=0.038), GORD (35.2% *versus* <20.4%; p=0.013) and were more likely to have had ≥1 exacerbations in the prior year (65.4% *versus* 40.8%; p<0.001) (supplementary material S10). They also reported significantly poorer asthma symptoms (ACQ-5 score 2.9 *versus* 1.2; p<0.001) and worse HRQoL (EQ-5D-5L utility score 0.67 *versus* 0.91; p<0.001) and significantly higher SABA usage when measured using both canisters dispensed (23 *versus* 2; p<0.001) and self-reported puffs per day (7.2 *versus* 1.6; p<0.001) (supplementary material S11). Our conclusions were unchanged when restricting our analysis to patients with at least 4 years follow-up after biologic initiation (supplementary material S12). Our conclusions did not change when using non-parametric methods to test for difference in GHG emissions pre- and post-biologic initiation (supplementary material S7).

## Discussion

Severe asthma was associated with substantial GHG emissions, which were largely driven by SABA use and emergency care. There was variation across patients, with those more deprived, younger in age, with more comorbidity and greater disease severity contributing higher GHG emissions. Biologic use was associated with a reduction in GHG emissions, which grew over time; however, the magnitude of this effect was modest and SABA use was relatively unchanged throughout the study. No relationship was found between GHG reduction and common measures of clinical response, with only modest decreases seen among those who could be defined as biologic super-responders or in disease remission.

Our study is novel, being the first to investigate the carbon footprint of severe asthma and to explore the change in GHG emissions after biologic initiation. Others have estimated GHG emissions in a broader asthma population managed within UK primary care and found mean GHG emissions ranging from 44 to 298 kg, with higher levels among those with greater asthma severity or poorer disease control [7]. Given the substantial baseline morbidity of the patients included in our study, the higher GHG emissions we have observed are to be expected. In line with our findings, previous studies have reported that SABA use and ED visits are important contributors to overall GHG emissions [7, 16]. Our finding of modest change in SABA use after biologic initiation has been demonstrated in other real-world studies. Within the UK, a recent study of 14 specialist centres reported that 54% of patients were taking at least 3 daily puffs at 1 year post-biologic initiation [11], while a single-centre study found the number of SABA doses decreased by 25% from 5.7 to 4.3 after 12 months of mepolizumab [24]. Similarly, data from the USA demonstrated a 27% decrease from 5.1 to 3.7 in SABA refills after benralizumab treatment [25]. Larger decreases of 45% from 13.0 to 7.1 puffs per day were observed in a study of Australian patients treated with mepolizumab, albeit baseline use was substantially higher than in our cohort [26].

Any successful environmental intervention in severe asthma must specifically target SABA use given its disproportionate contribution to GHG emissions. This is particularly important within the UK where SABA use is markedly higher than other European countries, which may be suggestive of an over-reliance on acute rather than preventative care [27]. Continued overuse of SABA post-biologic is likely to have important clinical consequences, and some have questioned whether reliever use should form an important component of disease remission [28]. Previous studies have identified that SABA users are psychologically attached to these medications due to the immediate symptom relief they offer [29, 30], suggesting that solutions are unlikely to be easy within severe asthma populations where SABA use is likely to have become entrenched. Widespread movement to “MART” (maintenance and reliever therapy) regimens [31] and the withdrawal of SABAs completely, in line with current Global Initiative for Asthma recommendations [32], would significantly reduce GHG emissions. However, it should be noted there is a paucity of evidence that such regimens are effective and safe in high-severity populations, such as the one studied here [33]. Switching from MDI to DPI devices when appropriate could lead to substantial GHG savings [17], while novel propellants such as HFC-152a and hydrofluoroolefins, which have a fraction of the global warming potential of current medications, are due to come to the market in 2025 and could have a major environmental impact [34].

Addressing the substantial carbon burden of severe asthma must be a priority if the NHS is to meet its “Net Zero” target by 2040 [4]. Although biologics have provided a paradigm shift in clinical outcomes among patients with severe asthma, our data suggest their effect on GHG emissions was modest, with little relationship found between clinical response and GHG change. Biologics did lead to a stark reduction in GHG emissions related to emergency healthcare utilisation; however, this was largely offset by increased outpatient visits. The GHG burden of outpatient appointments is likely to have receded in later years as an increasing proportion of patients self-administer their biologic at home. Although patients generally prefer biologic self-administration [35], the issue of which patients are likely to respond well to self-administration, and how often in-person clinical contact is required, remains an important research question.

Our results indicate that there was substantial residual morbidity post-biologic treatment for many patients, and that high symptom and exacerbation burden were associated with higher GHG emissions. Addressing this unmet need could lead to lower GHG emissions. Further work is required to elucidate the pathways underlying poor symptom control in biologic-treated populations to guide effective interventions. It should be noted that the patients included in our study were highly symptomatic with a substantial exacerbation and treatment burden prior to biologic initiation. It remains to be seen if intervention earlier in the treatment pathway, before patients become reliant on their reliever, could lead to improved clinical and environmental outcomes. Our findings that higher GHG emissions were associated with non-pulmonary factors such as deprivation, GORD and poor mental health also suggest that comorbidities may be important in driving GHG emissions. Others have advocated for a more holistic treatable traits approach in severe asthma which could improve disease control and consequently reduce GHG emissions [36].

The primary strength of the study lies in the data on which it is based. Linkage to high-quality data from specialist care provides information on patient clinical characteristics and asthma treatment, including variables such as type 2 biomarkers and spirometry which are not routinely available in other datasets. Linkage to population-level data on dispensing ensures that medications were picked up by patients, while data on emergency care use from across Northern Ireland ensures these healthcare contacts are appropriately captured without relying on patient recall. Our study has several potential limitations. Our single-arm design and univariable analysis may be prone to time-dependent confounders. Data were not available on primary care attendances. However, these have previously been estimated to contribute <3.3 kg per contact [7], so are unlikely to materially impact our findings. Similarly, data were available on only a small subset of comorbidities and it is possible that others (*e.g.* cardiovascular disease) could be associated with GHG emissions. Our analysis was restricted to only one centre which is ethnically homogeneous, and most patients received mepolizumab as their initial biologic, which may hinder the generalisability of our findings.

In conclusion, severe asthma is associated with a substantial level of GHG emissions, largely driven by SABA use and emergency care. Although biologic initiation was associated with a reduction in GHG emissions, the effect was modest and SABA use was relatively unchanged, even among those who could be classified as biologic super-responders or in disease remission. An improved understanding of the pulmonary and non-pulmonary factors that are driving residual GHG emissions in biologic-treated patients is required to develop effective interventions.

## Data Availability

The dataset for this project cannot be shared. Researchers seeking to access health-related datasets from Northern Ireland should contact the Northern Ireland Honest Broker Service (https://bso.hscni.net/directorates/digital/honest-broker-service) in the first instance.

## Supplementary material

**Please note:** supplementary material is not edited by the Editorial Office, and is uploaded as it has been supplied by the author. Supplementary material 01009-2024.SUPPLEMENT
