# Supplementary material for: Exploring the carbon footprint of severe asthma and change after biologic therapy initiation: an analysis of Northern Irish data
Source: ERJ Open Res. 2025 Jun 23;11(3):01009-2024. doi: 10.1183/23120541.01009-2024 (PMC12183723; doi:10.1183/23120541.01009-2024)
Supplement: Supplementary file 1 [file 01009-2024.SUPPLEMENT.pdf]

Online Supplement

Online Supplement 1: Supplementary methods

<u>Calculation of Green House Gas (GHG) emissions</u>

Medication-related emissions associated with use of β-agonists (BNF chapter: 3.1.1.1), inhaled corticosteroid (ICS; BNF chapter: 3.2), long-acting muscarinic antagonist (LAMA; BNF chapter: 3.1.2), theophylline (BNF chapter: 3.1.3), leukotriene receptor antagonist (LTRA; BNF chapter: 3.3.2), compound bronchodilators (BNF chapter: 3.1.4), oral corticosteroids (OCS; BNF chapter: 6.3.2) and biologics were included. GHG emissions were quantified using SimaPro life cycle assessment (LCA) software modelling resource and energy consumption data, Ecoinvent® datasets, certified published studies[1–3] and modelled estimates. Calculations for the carbon footprint considered emissions from the entire life cycle of inhalers, including the manufacture and transport of the inhaler device (e.g. pressurised metered dose inhaler [pMDI] or dry powder inhaler [DPI]), and end-of-life disposal. The study referred to the Intergovernmental Panel on Climate Change Fifth Assessment Report on Global Warming Potential of hydrofluoroalkanes (HFAs) for a 100-year time period to generate product LCAs.[4] Healthcare resource utilization (HCRU) related emissions were estimated using the methodology of the Sustainable Health Coalition's Sustainable Care Pathways Guidance.[5] Emissions related to asthma-related outpatient visits, accident & emergency (A&E) visits, and hospitalisations were estimated, and included an allowance for patient travel. No data were available on primary care consultations. Asthma hospitalisation was based on a primary diagnosis of asthma (ICD10 code: J45) while asthma-related ED visits were those where the diagnosis contained the word 'asthma'. We included outpatient visits with a 'Thoracic Medicine' or 'Thoracic Surgery' specialty

<u>Study Covariates</u>

The number of days hospitalisation for a patient in a single year was clipped at 60 days to prevent an undue influence of very-long hospital stays on population GHG estimates. The Northern Ireland Multiple Deprivation Measure (MDM) which provides a composite measure of relative deprivation across seven domains including income, employment, environment and crime. Self-reported SABA use was estimated using responses to the question six of the Asthma Control Questionnaire (ACQ): 'On average, during the past week, how many puffs of short-acting bronchodilator have you used each day' by taking the midpoint for each group (e.g. 3.5 puffs per day if the response was 3-4). For the highest group (>16 puffs) we conservatively assumed 17 puffs per day. Clinical data was associated with the 1-year annual review period if the study visit occurred between 6 and 18 months post biologic initiation.

Estimates of GHG emissions associated with medication use

Drug	Drug class	Device	GHG minimum range CO_2e (kg)/product	GHG maximum range CO_2e (kg)/product	References
Beclomethasone dipropionate	ICS	pMDI	12.61	19.28	[2, 3, 6, 7]
Beclomethasone dipropionate	ICS	DPI	0.42	0.84	[3, 6, 7]
Fluticasone propionate	ICS	pMDI	17.93	19.28	[2, 6]
Fluticasone propionate	ICS	DPI	0.51	0.90	[6–8]
Budesonide	ICS	pMDI	16.75	37.00	[2, 9]
Budesonide	ICS	DPI	0.33	1.63	[7, 9]
Ciclesonide	ICS	pMDI	8.40	12.61	[2]
Mometasone furoate	ICS	DPI	0.42	0.84	[10]
Fluticasone furoate/Vilanterol	ICS/LABA	DPI	0.78	0.90	[6]
Budesonide/Formoterol fumarate dihydrate	ICS/LABA	pMDI	12.61	34.40	[2, 9]
Budesonide/Formoterol fumarate dihydrate	ICS/LABA	DPI	0.33	1.05	[9]
Fluticasone propionate/Salmeterol xinafoate	ICS/LABA	pMDI	17.93	19.49	[2, 6]
Fluticasone propionate/Salmeterol xinafoate	ICS/LABA	DPI	0.90	0.90	[6]
Beclomethasone dipropionate/Formoterol fumarate dihydrate	ICS/LABA	pMDI	11.33	16.75	[2, 3]
Beclomethasone dipropionate/Formoterol fumarate dihydrate	ICS/LABA	DPI	0.51	0.92	[3, 7]
Fluticasone propionate/Formoterol fumarate dihydrate	ICS/LABA	pMDI	17.93	37.38	[2]
Salmeterol xinafoate	LABA	pMDI	12.61	26.94	[2, 6]
Salmeterol xinafoate	LABA	DPI	0.58	0.90	[6]
Formoterol fumarate dihydrate	LABA	pMDI	12.61	12.60	[2]
Formoterol fumarate dihydrate	LABA	DPI	0.26	0.78	[7–9]
Indacaterol maleate	LABA	DPI	0.78	0.78	[8]
Olodaterol hydrochloride	LABA	pMDI	16.30	16.30	[2, 10]
Tiotropium bromide	LAMA	pMDI	16.33	16.33	[2]

Drug	Drug class	Device	GHG minimum range CO_2e (kg)/product	GHG maximum range CO_2e (kg)/product	References
Tiotropium bromide	LAMA	DPI	0.74	0.78	[10]
Glycopyrronium bromide	LAMA	DPI	0.68	0.78	[8, 9]
Aclidinium bromide	LAMA	DPI	0.68	1.54	[9]
Umeclidinium bromide	LAMA	DPI	0.74	0.90	[6]
Vilanterol trifenatate/Umeclidinium bromide	LABA/LAMA	DPI	0.78	0.90	[6]
Aclidinium bromide/Formoterol fumarate dihydrate	LABA/LAMA	DPI	0.68	1.54	[9]
Olodaterol hydrochloride/Tiotropium bromide	LABA/LAMA	pMDI	16.33	16.33	[2, 10]
Indacaterol maleate/Glycopyrronium bromide	LABA/LAMA	DPI	0.40	0.78	[8]
Salbutamol sulphate	SABA	pMDI	8.87	28.30	[2, 6, 11]
Salbutamol sulphate	SABA	DPI	0.58	0.80	[6, 7]
Terbutaline sulphate	SABA	pMDI	37.00	37.00	[9]
Terbutaline sulphate	SABA	DPI	0.49	0.67	[9]
Vilanterol trifenatate/Umeclidinium bromide/Fluticasone furoate	ICS/LABA/LAMA	DPI	0.84	0.84	[6]
Theophylline	Xanthine	Oral	0.52	12.31	[9, 12]
Aminophylline	Xanthine	Oral	0.79	16.02	[8, 11]
Bambuterol hydrochloride	LABA	Oral	0.58	0.58	[9]
Montelukast	LTRA	Oral	0.23	0.47	[9]
Zafirlukast	LTRA	Oral	1.46	1.55	[9]
Prednisone	OCS	Oral	0.30	9.90	[6, 9, 12]
Prednisolone	OCS	Oral	0.30	10.00	[6, 9]
Omalizumab	Monoclonal antibodies	Subcutaneous	0.30	0.30	[9]
Mepolizumab	Monoclonal antibodies	Subcutaneous	0.30	0.30	[9]

Theophylline administered as a modified-release capsule (60, 125 or 250 mg), modified-release tablet (175, 200, 250, 300 or 400 mg), oral solution (10 or 12 mg/mL) or as a tablet (100 or 120 mg) in combination with ephedrine hydrochloride (11, 15 or 18.31 mg).
Aminophylline administered as a modified-release tablet (225 or 350 mg).
Bambuterol hydrochloride administered as a tablet (10 or 20 mg).
Montelukast administered as a tablet (10 mg), chewable tablet (4 or 5 mg) or granules (4 mg).
Zafirlukast administered as a tablet (20 mg).
Prednisone administered as a modified-release tablet (1, 2 or 5 mg) or tablet (1, 5 or 50 mg).
Prednisolone administered as a tablet (1, 2.5, 5, 10, 20, 25 or 30 mg).
The carbon footprint of oral medications was estimated for a pack of tablets for each dose.

CO_2e: carbon dioxide equivalent; DPI: dry powder inhaler; GHG: greenhouse gas; ICS: inhaled corticosteroid; LABA: long-acting β_2-agonist; LAMA: long-acting muscarinic antagonist; LTRA: leukotriene receptor antagonist; OCS: oral corticosteroid; pMDI: pressurised metered-dose inhaler; SABA: short-acting β_2-agonist.

Definition of HCRU carbon costs

**Visit type**	**Carbon cost**	**Assumptions**
Outpatient visit<br>(with return travel)	6.94 kg CO_2e	1.14 kg CO_2e (visit) + (2.9 × 2 kg CO_2e) (travel)
Inpatient hospitalisation<br>(via ED)	(0.97 × [37.9 kg CO_2e × number bed-days] + 4.83 kg CO_2e)	• 97% of bed-days were assumed to be accounted for by low-intensity bed-days (37.9 kg CO_2e/day) [13]<br>• UK average of high-intensity (ICU) stays at an average of 1.8 days, i.e., 89.39 CO_2e/high-intensity bed-day × 1.8 bed-days × 0.03 = 4.83 CO_2e
Inpatient hospitalisation<br>(not via ED)	(0.97 × [37.9 kg CO_2e × number bed-days] + 4.83 kg CO_2e) + 29.85 kg CO_2e (return travel)	Return travel: 50% ambulance cost (0.5 × 36 kg CO_2eq.) +<br>50% other transport cost (0.5 × 7.9 kg CO_2e) + 7.9 kg CO_2e travel cost for return
ED visit		
Arrival by ambulance	57.7 kg CO_2e	13.77 kg CO_2e (visit) + 36 kg CO_2e (travel by ambulance to hospital) +<br>7.9 kg CO_2e (non-emergency return)
Other modes of arrival	15.8 kg CO_2e	13.77 kg CO_2e (visit) + (2 × 7.9 kg CO_2e)<br>(for both travel to the hospital and return)
Unknown mode of arrival	39.4 kg CO_2e	13.77 kg CO_2e (visit) + (0.35 × 36 kg CO_2e) + (0.65 × 7.9 kg CO_2e) + 7.9 kg CO_2e for return<br>• Weighted CO_2e cost based on the proportion of ambulance and other modes of arrival in our data (35% ambulance and 65% other mode of arrival)

CO_2e: carbon dioxide equivalent; ED: emergency department; GP: general practitioner; HCRU: healthcare resource utilisation; ICU: intensive care unit; UK: United Kingdom.

Online Supplement 2: Patient flow diagram

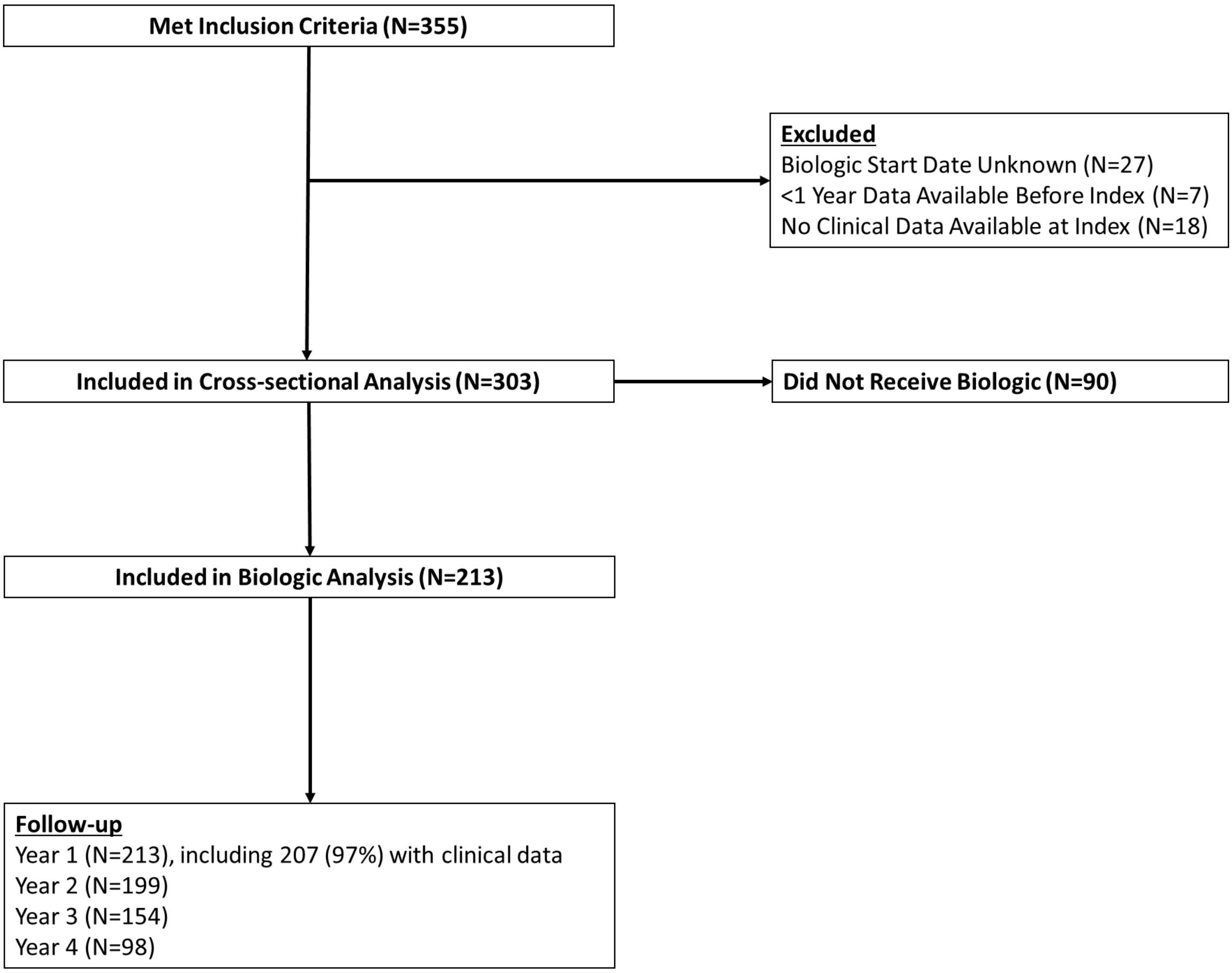

Online Supplement 3: Biologic use during the study

A – Biologic Use

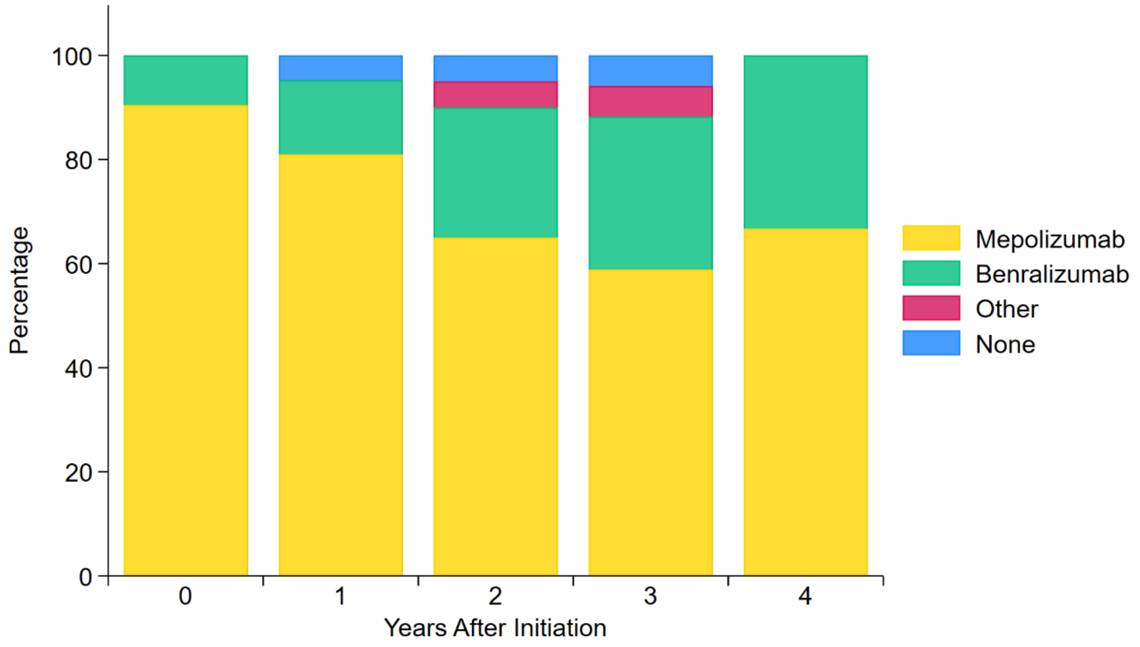

*counts are rounded to the nearest 10 to prevent disclosure

B – Biologic Continuation

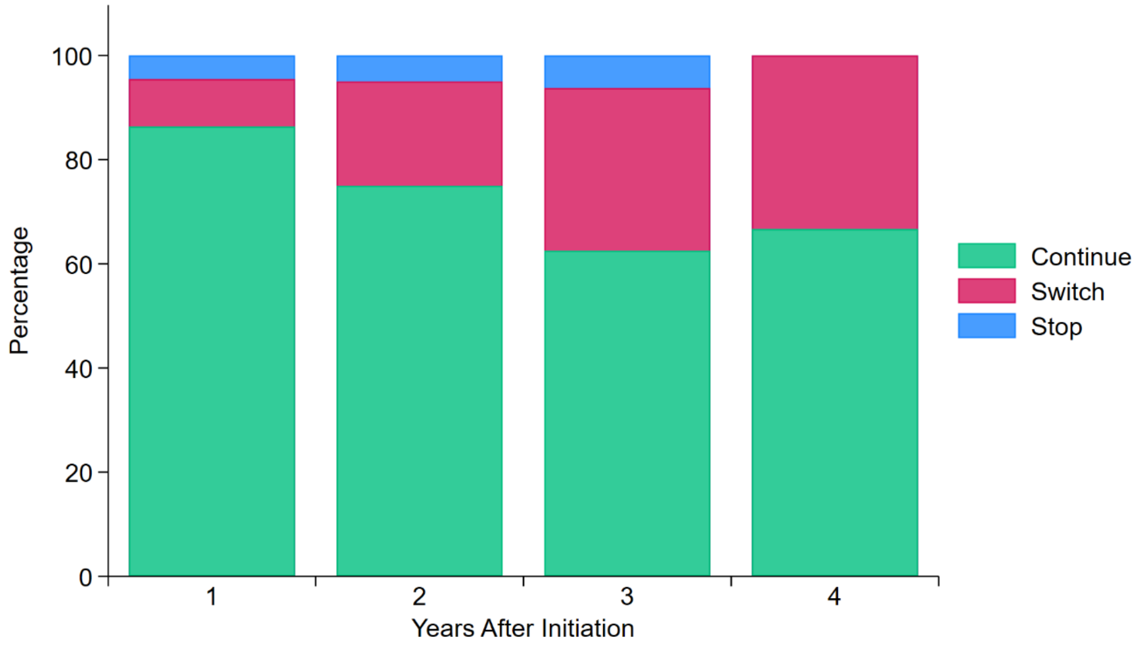

*counts are rounded to the nearest 10 to prevent disclosure

Online Supplement 4: Demographics, comorbidities and healthcare resource utilisation at index for patients progressing to biologics

**Number of Patients**	213
**GHG (kg CO_2e)**	462.2 (425.6)
**Demographics**	
Female	127 (59.6%)
Age (Years)	54.5 (12.8)
Asthma Duration (Years)	27.1 (16.3)
Caucasian	213 (100.0%)
Deprivation Tertile	
1 (Most)	65 (30.8%)
2	75 (35.5%)
3 (Least)	71 (33.6%)
BMI (kg/m^2)	31.2 (6.8)
Obese	111 (52.6%)
Ever Smoker	85 (40.1%)
Atopic Disease	61 (46.6%)
**Comorbidities**	
Depression or Anxiety	36 (16.9%)
GERD	49 (23.0%)
Nasal Polyps	44 (20.7%)
**HCRU (Last Year)**	
Exacerbations	4 (3,6)
Any Asthma ED Attendance	41 (19.2%)
Any Asthma Hospitalisation	46 (21.6%)
Outpatients Visits	9 (6,13)

Data are presented as n, mean±SD, median (interquartile range) or n (%), unless otherwise stated. BMI: body mass index; ED: emergency department; GHG: Greenhouse Gas emissions; HCRU: healthcare resource utilisation

Online Supplement 5: Clinical measures and asthma medications at index for patients progressing to biologics

**Number of Patients**	213
**Lung Function**	
FEV_1 (%)	69.2 (20.7)
FVC (%)	85.9 (17.7)
FEV_1/FVC	63.8 (12.7)
**Patient Reported Outcomes**	
ACQ-5 Score	2.9 (1.4)
EuroQoL Utility	0.71 (0.45,0.92)
**T2 Biomarkers**	
BEC (N/10^9L)	0.34 (0.14,0.56)
Highest BEC (N/10^9L)	0.74 (0.60,1.06)
FeNO (ppb)	33 (20,57)
IgE (IU/mL)	119 (48,332)
**Asthma Medications**	
Daily ICS Dose (BDP equivalent [ug])	2000 (2000,2000)
SABA Inhalers	10 (4,18)
Daily SABA Puffs	5.5 (4.6)
pMDI SABA	144 (73.8%)
Maintenance OCS	153 (72.2%)
Daily Dose (mg)	10 (8,10)
LAMA	54 (37.5%)
Theophylline	54 (37.8%)
LTRA	71 (49.3%)
Macrolides	19 (9.0%)
Nebuliser	32 (22.4%)

Data are presented as n, mean±SD, median (interquartile range) or n (%), unless otherwise stated.

ACQ-5: Asthma Control Questionnaire-5; BDP: beclometasone dipropionate; BEC: blood eosinophil count; FeNO: exhaled nitric oxide fraction; FEV_1: forced expiratory volume in 1 second; FVC: forced vital capacity; ICS: inhaled corticosteroids; IgE: immunoglobulin E; LAMA: long-acting muscarinic antagonists; LTRA: leukotriene receptor antagonists; OCS: oral corticosteroids; SABA: short-acting β_2-agonist

Online Supplement 6: Healthcare resource utilisation (HCRU), clinical measures and asthma medications at annual review (post-biologic initiation)

**Number of Patients**	207
**HCRU (Last Year)**	
Any Asthma Exacerbations	120 (59.1%)
Any Asthma ED Attendance	21 (10.1%)
Any Asthma Hospitalisation	16 (7.7%)
**Lung Function**	
FEV_1 (%)	73.0 (21.1)
FVC (%)	89.1 (17.7)
FEV_1/FVC	64.7 (13.3)
**Patient Reported Outcomes**	
ACQ-5 Score	2.1 (1.4)
EuroQoL Utility	0.75 (0.49,0.94)
**T2 Biomarkers**	
BEC (N/10^9L)	0.06 (0.03,0.10)
FeNO (ppb)	37 (21,63)
**Asthma Medications**	
SABA Inhalers	8 (3,17)
Daily SABA Puffs	4.2 (4.5)
Maintenance OCS	91 (44.2%)
**Response**	
Δ ACQ-5 Score	-0.6 (-1.4,0.0)
Δ Exacerbations	-3 (-5,-1)
Δ FEV_1 (mL)	50 (-190,300)
Reduced OCS Dose	129 (87.8%)
Δ Maintenance OCS Dose (mg)	-6.0 (-10.0,-5.0)
Δ Daily SABA Puffs	0.0 (-3.5,0.0)
**Composite Response**	
Super Responder	34 (19.4%)
Remission	20 (10.6%)

Data are presented as n, mean (SD), median (interquartile range) or n (%), unless otherwise stated. ACQ: Asthma Control Questionnaire; BEC: blood eosinophil count; ED: emergency department; FeNO: fractional exhaled nitric oxide; FEV_1: forced expiratory volume in one second; FVC: forced vital capacity; HCRU: healthcare resource utilisation; OCS: oral corticosteroid; SABA: short-acting β_2-agonist

Super-response was defined as ACQ-6 improvement ≥0.5 (or well controlled [ACQ-6 ≤0.75] at annual review), and exacerbation reduction ≥50% (or no exacerbations in the 12 months prior to annual review), and unscheduled care reduction ≥50% (or no unscheduled care in the 12 months prior to annual review), FEV_1 improvement ≥100 mL, and OCS dose reduction ≥50% (or not a mOCS user at annual review). Clinical remission was defined as no evidence of poor symptom control [ACQ5≤1.5]), no exacerbations in the previous 12 months and no maintenance OCS at the time of first annual review.

Online Supplement 7: GHGs by category and year[a]

Medication Class	Period	N	Mean CO_2e (kg)	Δ Baseline	P-value	
					T-test	Sign-rank
SABA	0	213	234.3 (310.4)			
	1	213	228.2 (313.0)	-6.1 (138.0)	0.518	0.428
	2	199	229.5 (360.9)	-2.0 (175.9)	0.873	0.544
	3	154	228.5 (400.1)	-12.7 (217.7)	0.471	0.915
	4	98	246.3 (546.1)	13.9 (276.1)	0.620	0.651
ICS / LABA	0	213	43.2 (99.9)			
	1	213	39.0 (90.7)	-4.2 (54.9)	0.264	0.944
	2	199	31.4 (74.9)	-10.0 (70.8)	0.048	0.397
	3	154	30.3 (68.9)	-8.4 (77.1)	0.180	0.041
	4	98	33.0 (80.8)	3.1 (49.1)	0.533	0.047
LAMA	0	213	3.0 (4.9)			
	1	213	3.5 (5.2)	0.5 (3.0)	0.011	0.014
	2	199	3.6 (5.5)	0.9 (3.5)	0.001	<0.001
	3	154	3.8 (6.8)	1.4 (3.8)	<0.001	<0.001
	4	98	4.6 (7.5)	1.8 (4.3)	<0.001	<0.001
Theophylline	0	213	9.4 (15.9)			
	1	213	8.6 (14.0)	-0.8 (8.1)	0.175	0.712
	2	199	9.7 (15.4)	0.3 (10.4)	0.725	0.287
	3	154	9.9 (16.1)	0.6 (10.3)	0.497	0.013
	4	98	9.2 (16.0)	0.4 (13.4)	0.789	0.221
LTRA	0	213	2.3 (2.8)			
	1	213	2.2 (2.5)	-0.1 (1.5)	0.320	0.638
	2	199	2.1 (2.4)	-0.1 (2.2)	0.589	0.898
	3	154	2.2 (2.5)	0.0 (2.5)	0.814	0.075
	4	98	1.9 (2.5)	-0.1 (2.8)	0.812	0.322
OCS	0	213	12.9 (8.9)			
	1	213	8.4 (8.0)	-4.4 (7.0)	<0.001	<0.001
	2	199	6.6 (7.9)	-6.4 (8.8)	<0.001	<0.001
	3	154	5.5 (6.5)	-7.7 (8.2)	<0.001	<0.001
	4	98	4.3 (6.0)	-9.8 (10.4)	<0.001	<0.001
Biologics	0	213	0.0 (0.0)			
	1	213	3.3 (0.6)	3.3 (0.6)	<0.001	<0.001
	2	199	3.1 (1.0)	3.1 (1.0)	<0.001	<0.001
	3	154	3.0 (1.3)	3.0 (1.3)	<0.001	<0.001
	4	98	3.1 (1.2)	3.1 (1.2)	<0.001	<0.001
Other Meds	0	213	2.5 (20.4)			
	1	213	2.9 (24.1)	0.4 (6.4)	0.341	0.078
	2	199	2.9 (26.1)	0.2 (7.5)	0.664	0.032
	3	154	1.9 (11.6)	-1.5 (13.4)	0.157	0.128
	4	98	1.4 (8.8)	-1.7 (19.9)	0.392	0.520
ED / Inpatients	0	213	84.6 (237.1)			
	1	213	25.3 (104.0)	-59.3 (223.8)	<0.001	<0.001
	2	199	32.6 (186.2)	-47.5 (228.1)	0.004	<0.001
	3	154	19.0 (144.8)	-74.7 (238.7)	<0.001	<0.001
	4	98	7.4 (40.8)	-68.1 (200.8)	0.001	<0.001
Outpatients	0	213	70.1 (41.0)			
	1	213	112.8 (32.1)	42.7 (47.6)	<0.001	<0.001

	2	199	87.1 (42.3)	16.0 (53.2)	<0.001	<0.001
	3	154	67.7 (45.2)	-4.3 (60.4)	0.375	0.412
	4	98	56.9 (41.7)	-15.0 (52.3)	0.005	0.007
Overall Medications	0	213	307.5 (333.7)			
	1	213	296.1 (335.6)	-11.4 (158.8)	0.298	0.148
	2	199	288.9 (373.3)	-14.0 (195.1)	0.312	0.713
	3	154	285.1 (409.5)	-25.3 (235.8)	0.185	0.327
	4	98	303.9 (559.3)	10.7 (290.7)	0.717	0.784
Overall HCRU	0	213	154.8 (245.5)			
	1	213	138.2 (113.0)	-16.6 (231.7)	0.297	<0.001
	2	199	119.7 (201.2)	-31.4 (232.2)	0.058	0.927
	3	154	86.6 (162.2)	-79.0 (247.6)	<0.001	0.001
	4	98	64.3 (62.1)	-83.1 (210.5)	<0.001	<0.001
Overall	0	213	462.2 (425.6)			
	1	213	434.3 (360.1)	-28.0 (285.5)	0.154	0.845
	2	199	408.6 (424.9)	-45.5 (306.1)	0.037	0.125
	3	154	371.8 (437.6)	-104.3 (346.3)	<0.001	<0.001
	4	98	368.2 (571.4)	-72.4 (351.6)	0.044	0.004

[a] Study period 0 represents year prior to biologic initiation.

CO_2e: carbon dioxide equivalent; ED: emergency department; GHG: greenhouse case; HCRU: Health care resource utilisation; ICS: inhaled corticosteroids; LABA: long-acting β_2 agonist; LAMA: long-acting muscarinic antagonists; LTRA: leukotriene receptor antagonists; OCS: oral corticosteroids; SABA: short-acting $\beta_2$agonist

Online Supplement 8: Relative change in GHGs by markers of clinical response

Exacerbations (N=181, rho=-0.01, p=0.855)

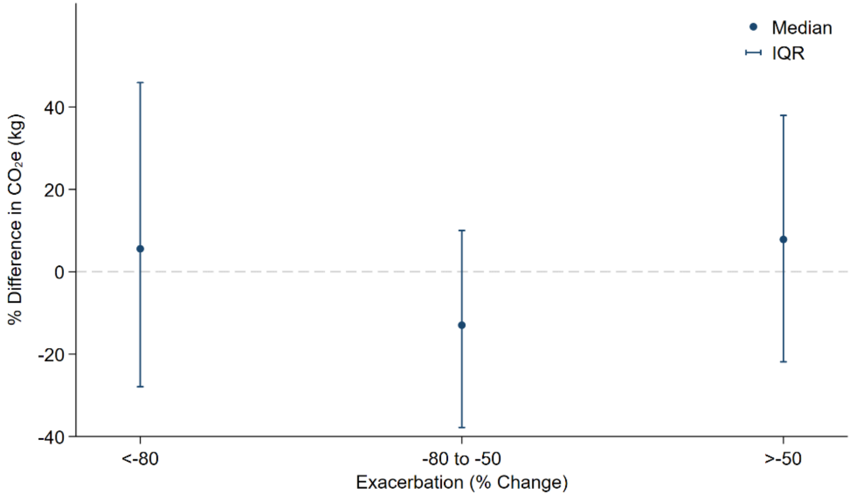

ACQ5 (N=183, rho=0.03, p=0.683)

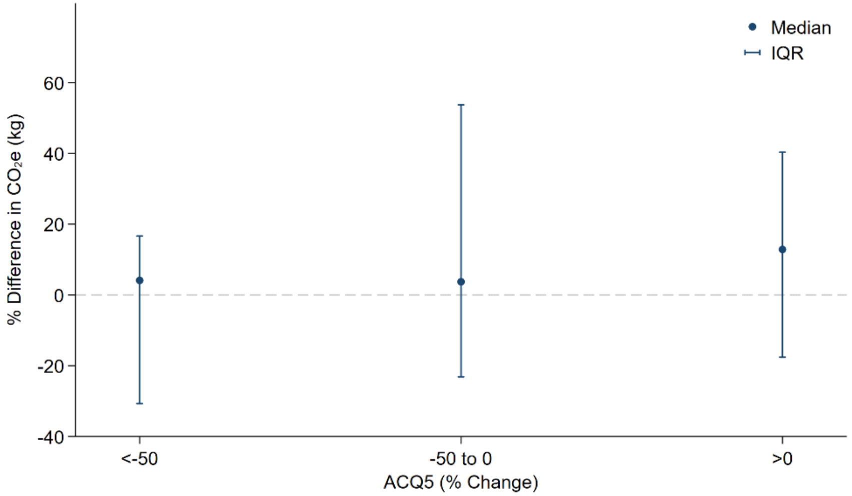

FEV$_1$ (N=202, rho=0.00, p=0.990)

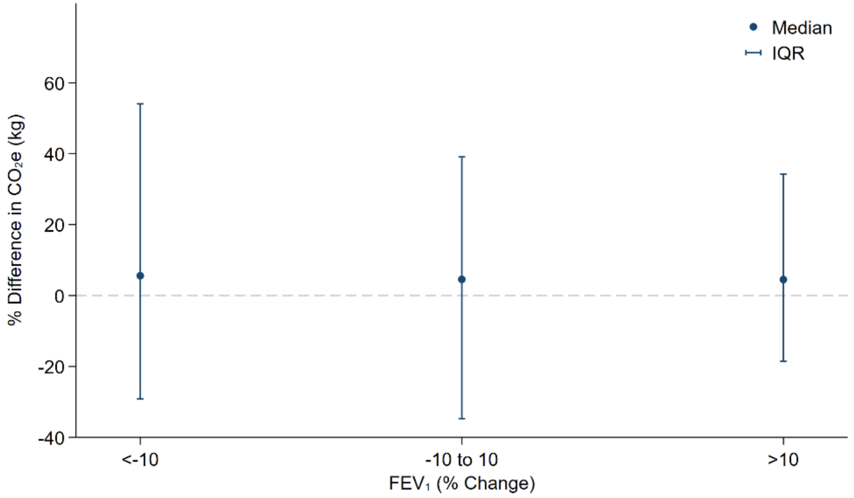

EuroQoL (N=173, rho=0.01, p=0.944)

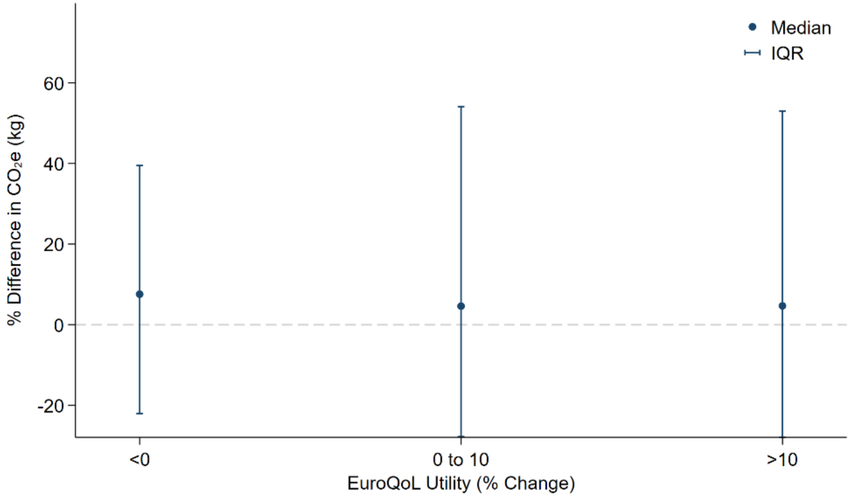

ACQ: Asthma Control Questionnaire; CO_2e: carbon dioxide equivalent; EuroQoL: European Quality of Life Assessment Questionnaire; FEV_1: forced expiratory volume in one second;

Online Supplement 9 GHGs in the year following index by category and composite response status

Carbon Category	No			Yes			P-value	
	N	Mean CO_2e (kg, SD)	Δ Baseline	N	Mean CO_2e (kg)	Δ Baseline	T-test	MW U
**Super Response**								
SABA	141	227.7 (337.2)	-8.6 (134.0)	34	185.8 (194.8)	-18.4 (112.4)	0.694	0.914
ICS / LABA	141	35.7 (89.8)	-2.4 (52.8)	34	71.2 (123.8)	3.6 (73.2)	0.584	0.693
LAMA	141	3.9 (5.4)	0.7 (3.1)	34	3.3 (4.9)	0.6 (2.5)	0.783	0.344
Theophylline	141	8.7 (13.9)	-0.4 (6.1)	34	8.7 (14.5)	0.6 (5.0)	0.373	0.581
LRTA	141	2.2 (2.5)	-0.0 (1.6)	34	2.1 (2.5)	-0.1 (1.4)	0.980	0.144
OCS	141	8.5 (7.1)	-3.9 (6.7)	34	4.9 (4.6)	-6.3 (6.6)	0.056	0.113
Biologics	141	3.4 (0.6)	3.4 (0.6)	34	3.3 (0.6)	3.3 (0.6)	0.812	0.746
Other Meds	141	3.1 (27.7)	0.1 (5.9)	34	3.7 (19.5)	0.7 (4.3)	0.566	0.845
ED / Inpatients	141	33.3 (120.7)	-57.4 (240.6)	34	0.0 (0.0)	-37.8 (96.3)	0.642	0.819
Outpatients	141	113.7 (30.8)	41.2 (47.3)	34	105.5 (25.4)	44.5 (35.8)	0.703	0.780
Overall Meds	141	293.3 (353.5)	-11.1 (155.7)	34	283.1 (267.6)	-15.9 (138.1)	0.869	0.955
Overall HCRU	141	147.0 (129.4)	-16.2 (249.8)	34	105.5 (25.4)	6.7 (106.4)	0.602	0.925
Overall	141	440.3 (382.2)	-27.3 (299.1)	34	388.6 (275.9)	-9.2 (157.3)	0.733	0.806
**Remission**								
SABA	169	246.2 (330.2)	-7.3 (134.9)	20	98.8 (131.9)	-29.8 (147.1)	0.485	0.224
ICS / LABA	169	39.0 (92.1)	-2.2 (57.2)	20	49.8 (115.6)	-9.1 (57.6)	0.614	0.759
LAMA	169	3.8 (5.2)	0.6 (3.1)	20	1.8 (4.7)	0.9 (2.8)	0.680	0.948
Theophylline	169	8.9 (14.2)	-1.0 (8.6)	20	6.5 (10.9)	1.0 (5.9)	0.319	0.398
LTRA	169	2.3 (2.5)	-0.0 (1.6)	20	1.5 (2.0)	-0.2 (1.1)	0.683	0.198
OCS	169	9.2 (8.4)	-4.5 (7.2)	20	2.6 (2.0)	-5.7 (4.5)	0.450	0.302
Biologics	169	3.3 (0.6)	3.3 (0.6)	20	3.4 (0.6)	3.4 (0.6)	0.613	0.403
Other Meds	169	3.3 (26.7)	0.2 (5.7)	20	0.0 (0.0)	-0.0 (0.1)	0.863	0.740
ED / Inpatients	169	27.8 (110.9)	-60.4 (226.9)	20	0.0 (0.0)	-9.5 (30.8)	0.319	0.360
Outpatients	169	115.1 (32.6)	45.9 (45.6)	20	98.2 (27.8)	15.6 (44.1)	0.005	0.008
Overall Meds	169	316.0 (351.1)	-11.0 (158.7)	20	164.4 (167.7)	-39.6 (150.8)	0.444	0.240
Overall HCRU	169	142.8 (119.6)	-14.5 (235.6)	20	98.2 (27.8)	6.1 (49.0)	0.698	0.132
Overall	169	458.9 (374.5)	-25.5 (287.0)	20	262.6 (174.5)	-33.6 (155.0)	0.902	0.272

CO_2e: carbon dioxide equivalent; ED: emergency department; HCRU: Health care resource utilisation; ICS: inhaled corticosteroids; LABA: long-acting $\beta_2$agonist; LAMA: long-acting muscarinic antagonists; LTRA: leukotriene receptor antagonists; MW-U: Mann-Whitney U; OCS: oral corticosteroids; SABA: short-acting $\beta_2$agonist; SD: standard deviation

Super-response was defined as ACQ-6 improvement ≥0.5 (or well controlled [ACQ-6 ≤0.75] at annual review), and exacerbation reduction ≥50% (or no exacerbations in the 12 months prior to annual review), and unscheduled care reduction ≥50% (or no unscheduled care in the 12 months prior to annual review), FEV_1 improvement ≥100 mL, and OCS dose reduction ≥50% (or not a mOCS user at annual review). Clinical remission was defined as no evidence of poor symptom control [ACQ5≤1.5]), no exacerbations in the previous 12 months and no maintenance OCS at the time of first annual review.

Online Supplement 10: Demographics, comorbidities and healthcare resource utilisation 1-year post-biologic initiation by GHG quartile

	All Patients	GHG Quartile				P-value
		1 (Lowest)	2	3	4 (Highest)	
**Number of Patients**	207	49	51	53	54	
**GHG (kg CO_2e)**	431.8 (361.5)	137.2 (29.0)	249.3 (43.8)	418.1 (72.7)	885.1 (422.3)	<0.001
**Demographics**						
Female	123 (59.4%)	27 (55.1%)	24 (47.1%)	37 (69.8%)	35 (64.8%)	0.084
Age (Years)	55.4 (12.9)	58.2 (10.8)	56.0 (13.9)	53.6 (13.9)	54.0 (12.4)	0.253
Asthma Duration (Years)	27.0 (16.3)	23.7 (13.8)	28.6 (19.3)	27.5 (15.6)	28.1 (16.3)	0.444
Caucasian	207 (100.0%)	49 (100.0%)	51 (100.0%)	53 (100.0%)	54 (100.0%)	
Deprivation Tertile						0.039
1 (Most)	62 (30.2%)	<20.4%	12 (24.5%)	20 (37.7%)	22 (40.7%)	
2	74 (36.1%)	17 (34.7%)	19 (38.8%)	21 (39.6%)	17 (31.5%)	
3 (Least)	69 (33.7%)	24 (49.0%)	18 (36.7%)	12 (22.6%)	15 (27.8%)	
BMI (kg/m^2)	30.9 (6.6)	30.4 (7.0)	29.9 (6.8)	30.5 (6.2)	32.6 (6.5)	0.163
Obese	99 (49.7%)	22 (48.9%)	20 (40.0%)	26 (50.0%)	31 (59.6%)	0.268
Ever Smoker	82 (39.8%)	13 (26.5%)	24 (48.0%)	17 (32.1%)	28 (51.9%)	0.022
**Comorbidities**						
Depression or Anxiety	34 (16.4%)	<20.4%	<19.6%	<18.9%	15 (27.8%)	0.038
GERD	48 (23.2%)	<20.4%	13 (25.5%)	12 (22.6%)	19 (35.2%)	0.013
Nasal Polyps	43 (20.8%)	15 (30.6%)	10 (19.6%)	<18.9%	<18.5%	0.268
**HCRU (Last Year)**						
Any Asthma Exacerbations	120 (59.1%)	20 (40.8%)	22 (44.9%)	>81.1%	34 (65.4%)	<0.001
Any Asthma ED Attendance	21 (10.1%)	<20.4%	<19.6%	<18.9%	<18.5%	0.244
Any Asthma Hospitalisation	16 (7.7%)	<20.4	<19.6%	<18.9%	10 (18.5%)	0.001
Outpatients Visits	16 (13, 19)	14 (12, 16)	17 (14, 20)	15 (12, 17)	17 (15, 20)	<0.001

Data are presented as n, mean (SD), median (interquartile range) or n (%), unless otherwise stated.

BMI: body mass index; CO2e: carbon dioxide equivalent; ED: emergency department; GERD: gastro-oesophageal reflux disease; GHG: greenhouse gases ; HCRU: healthcare resource utilization

Online Supplement 11: Clinical measures and asthma medications 1 year post-biologic initiation by GHG quartile

	All Patients	GHG Quartile				P-value
		1 (Lowest)	2	3	4 (Highest)	
**Number of Patients**	207	49	51	53	54	
**GHG (kg CO_2e)**	322 (184,566)	137 (123,162)	251 (210,291)	417 (359,469)	783 (635,991)	<0.001
**Lung Function**						
FEV_1 (%)	73.0 (21.1)	76.5 (20.9)	76.4 (19.6)	70.6 (21.6)	68.9 (21.6)	0.149
FVC (%)	89.1 (17.7)	92.4 (17.0)	91.6 (14.8)	85.4 (18.1)	87.5 (19.8)	0.154
FEV_1/FVC	64.7 (13.3)	65.2 (12.6)	66.2 (14.1)	64.8 (12.7)	62.7 (13.7)	0.593
**Patient Reported Outcomes**						
ACQ-5 Score	2.1 (1.4)	1.2 (1.1)	1.9 (1.1)	2.3 (1.5)	2.9 (1.3)	<0.001
EuroQoL Utility	0.75 (0.49,0.94)	0.91 (0.73,1.00)	0.79 (0.68,0.93)	0.70 (0.30,0.92)	0.67 (0.27,0.83)	<0.001
**T2 Biomarkers**						
BEC ($N/10^9L$)	0.06 (0.03,0.10)	0.06 (0.02,0.10)	0.06 (0.03,0.09)	0.06 (0.02,0.10)	0.06 (0.03,0.11)	0.614
FeNO (ppb)	37 (21,63)	29 (18,42)	44 (25,70)	42 (26,62)	31 (21,54)	0.048
**Asthma Medications**						
SABA Inhalers	8 (3,17)	2 (0,4)	5 (3,8)	10 (7,14)	23 (16,35)	<0.001
Daily SABA Puffs	4.2 (4.5)	1.6 (2.2)	2.5 (3.2)	5.1 (4.3)	7.2 (5.1)	<0.001
Maintenance OCS	91 (44.2%)	21 (42.9%)	22 (43.1%)	21 (39.6%)	27 (50.9%)	0.685

Data are presented as n, mean (SD), median (interquartile range) or n (%), unless otherwise stated.

ACQ-5: Asthma Control Questionnaire-5; BEC: blood eosinophil count; CO_2e: carbon dioxide equivalent; FeNO: exhaled nitric oxide fraction; FEV_1: forced expiratory volume in one second; FVC: forced vital capacity; GHG: greenhouse gases; OCS: oral corticosteroids; SABA: short-acting β_2 agonist

Online Supplement 12: GHGs by category and year[a], restricted to patients with at least four years follow-up after biologic initiation

Medication Class	Period	N	Mean CO_2e (kg)	Δ Baseline	P-value	
					T-test	Sign-rank
SABA	0	98	232.4 (371.0)			
	1	98	236.9 (394.7)	4.5 (149.2)	0.764	0.898
	2	98	253.8 (457.9)	21.4 (196.2)	0.282	0.042
	3	98	243.8 (479.8)	11.4 (217.8)	0.606	0.231
	4	98	246.3 (546.1)	13.9 (276.1)	0.620	0.651
ICS / LABA	0	98	29.9 (81.4)			
	1	98	32.8 (77.9)	2.9 (39.3)	0.470	0.318
	2	98	33.0 (73.2)	3.1 (45.4)	0.501	0.030
	3	98	30.4 (67.5)	0.5 (46.5)	0.915	0.010
	4	98	33.0 (80.8)	3.1 (49.1)	0.533	0.047
LAMA	0	98	2.7 (5.5)			
	1	98	3.2 (5.0)	0.5 (2.7)	0.066	0.033
	2	98	3.5 (5.7)	0.8 (3.6)	0.034	0.003
	3	98	4.4 (7.8)	1.6 (4.3)	<0.001	<0.001
	4	98	4.6 (7.5)	1.8 (4.3)	<0.001	<0.001
Theophylline	0	98	8.9 (16.0)			
	1	98	7.4 (12.0)	-1.4 (10.8)	0.201	0.986
	2	98	8.6 (13.1)	-0.2 (12.7)	0.852	0.263
	3	98	9.6 (15.4)	0.8 (10.2)	0.455	0.012
	4	98	9.2 (16.0)	0.4 (13.4)	0.789	0.221
LTRA	0	98	2.0 (3.4)			
	1	98	1.7 (2.5)	-0.2 (2.0)	0.236	0.367
	2	98	1.8 (2.4)	-0.1 (2.9)	0.662	0.778
	3	98	2.0 (2.4)	-0.0 (2.9)	0.990	0.187
	4	98	1.9 (2.5)	-0.1 (2.8)	0.812	0.322
OCS	0	98	14.2 (10.4)			
	1	98	8.8 (8.4)	-5.4 (6.5)	<0.001	<0.001
	2	98	7.4 (7.6)	-6.7 (9.6)	<0.001	<0.001
	3	98	5.6 (7.0)	-8.5 (8.8)	<0.001	<0.001
	4	98	4.3 (6.0)	-9.8 (10.4)	<0.001	<0.001
Biologics	0	98	0.0 (0.0)			
	1	98	3.6 (0.3)	3.6 (0.3)	<0.001	<0.001
	2	98	3.3 (0.8)	3.3 (0.8)	<0.001	<0.001
	3	98	3.2 (1.1)	3.2 (1.1)	<0.001	<0.001
	4	98	3.1 (1.2)	3.1 (1.2)	<0.001	<0.001
Other Meds	0	98	3.2 (28.0)			
	1	98	3.8 (33.0)	0.6 (5.2)	0.242	0.448
	2	98	4.0 (36.3)	0.9 (8.3)	0.307	0.121
	3	98	1.7 (13.5)	-1.4 (14.9)	0.343	0.080
	4	98	1.4 (8.8)	-1.7 (19.9)	0.392	0.520
ED / Inpatients	0	98	75.5 (202.2)			
	1	98	22.6 (77.0)	-52.8 (187.3)	0.006	0.004
	2	98	22.3 (81.0)	-53.2 (198.4)	0.009	0.003
	3	98	7.3 (45.8)	-68.2 (199.8)	0.001	<0.001
	4	98	7.4 (40.8)	-68.1 (200.8)	0.001	<0.001
Outpatients	0	98	71.9 (39.3)			

	1	98	119.0 (29.0)	47.1 (44.7)	<0.001	<0.001
	2	98	94.5 (32.5)	22.5 (44.9)	<0.001	<0.001
	3	98	71.9 (40.2)	-0.1 (57.5)	0.990	0.927
	4	98	56.9 (41.7)	-15.0 (52.3)	0.005	0.007
Overall Medications	0	98	293.2 (381.0)			
	1	98	298.3 (409.5)	5.1 (164.8)	0.761	0.970
	2	98	315.6 (469.6)	22.4 (216.9)	0.310	0.078
	3	98	300.7 (488.2)	7.5 (227.7)	0.745	0.624
	4	98	303.9 (559.3)	10.7 (290.7)	0.717	0.784
Overall HCRU	0	98	147.4 (214.3)			
	1	98	141.7 (84.3)	-5.7 (198.7)	0.776	0.004
	2	98	116.7 (95.4)	-30.7 (210.0)	0.151	0.353
	3	98	79.2 (62.7)	-68.2 (210.6)	0.002	0.045
	4	98	64.3 (62.1)	-83.1 (210.5)	<0.001	<0.001
Overall	0	98	440.6 (443.4)			
	1	98	440.0 (420.6)	-0.7 (256.8)	0.980	0.443
	2	98	432.3 (479.1)	-8.3 (299.8)	0.785	0.495
	3	98	379.9 (493.2)	-60.7 (305.0)	0.052	0.072
	4	98	368.2 (571.4)	-72.4 (351.6)	0.044	0.004

[a] Study period 0 represents year prior to biologic initiation.

CO_2e: carbon dioxide equivalent; ED: emergency department; GHG: greenhouse case; HCRU: Health care resource utilisation; ICS: inhaled corticosteroids; LABA: long-acting β_2 agonist; LAMA: long-acting muscarinic antagonists; LTRA: leukotriene receptor antagonists; OCS: oral corticosteroids; SABA: short-acting $\beta_2$agonist

References

1 Wilkinson AJK, Braggins R, Steinbach I, *et al.* Costs of switching to low global warming potential inhalers. An economic and carbon footprint analysis of NHS prescription data in England. *BMJ Open* England; 2019; 9: e028763.

2 Jeswani H, Azapagic A. Life cycle environmetal impacts of inhalers. *J Cleaner Production* 2019; 237: 117733.

3 Panigone S, Sandri F, Ferri R, *et al.* Environmental impact of inhalers for respiratory diseases: decreasing the carbon footprint while preserving patient-tailored treatment. *BMJ Open Respir Res* England; 2020; 7.

4 Intergovernmental Panel on Climate Change. Global warming potential values. 2014; .

5 Sustainable Healthcare Coalition, Informing and inspiring global health systems to make the transition to sustainability. . Available from: https://shcoalition.org/. [last accessed 8th July 2024]

6 Janson C, Menzies-Gow A, Nan C, *et al.* SABINA: An Overview of Short-Acting β(2)-Agonist Use in Asthma in European Countries. *Adv Ther* United States; 2020; 37: 1124–1135.

7 Borenius K, Vartiainen V, Takala A, *et al.* Life cycle assesment (LCA) and cradle-to-grave (CTG) carbon footpring of a multidose reservoir dry powder inhaler. *ERS virtual congress 2020 - asthma inhalers: devices and adherence studies* 2020; E-poster session: 3183.

8 Novartis. Case study: Breezhaler carbon footprint. . Available from: https://www.novartis.com/esg/environmental-sustainability/climate/case-study-breezhaler-carbon-footprint. [Last accessed 8th July 2024]

9 AstraZeneca life cycle assessment internal reports. *Data on file*.

10 Hänsel M, Bambach T, Wachtel H. Reduced Environmental Impact of the Reusable Respimat(®) Soft Mist™ Inhaler Compared with Pressurised Metered-Dose Inhalers. *Adv Ther* United States; 2019; 36: 2487–2492.

11 Sellers WFS. Asthma pressurised metered dose inhaler performance: propellant effect studies in delivery systems. *Allergy Asthma Clin Immunol* England; 2017; 13: 30.

12 Sherman J, Le C, Lamers V, *et al.* Life cycle greenhouse gas emissions of anesthetic drugs. *Anesth Analg* United States; 2012; 114: 1086–1090.

13 Wilkinson AJK, Maslova E, Janson C, *et al.* Greenhouse gas emissions associated with suboptimal asthma care in the UK: the SABINA healthCARe-Based envirONmental cost of treatment (CARBON) study. *Thorax* England; 2024; : thorax-2023-220259.
